# Melanization as unfavorable factor in amelanotic melanoma cell biology

**DOI:** 10.1007/s00709-021-01613-5

**Published:** 2021-01-27

**Authors:** A. Skoniecka, M. Cichorek, A. Tyminska, I. Pelikant-Malecka, J. Dziewiatkowski

**Affiliations:** 1 Embryology Department, Medical University of Gdansk, Ul. Debinki 1 St, 80-211, Gdansk, Poland; 2 Department of Medical Laboratory Diagnostics-Biobank, Medical University of Gdansk, 80-211 Gdansk, Poland; 3 Biobanking and Biomolecular Resources Research Infrastructure Poland (BBMRI.PL), 80-211 Gdansk, Poland; 4 Department of Anatomy and Neurobiology, Medical University of Gdansk, Debinki 1 St, 80-211, Gdansk, Poland

**Keywords:** Amelanotic melanoma, Melanoma, Apoptosis, Melanization, L-tyrosine, Melanin

## Abstract

The biology of three amelanotic melanoma cell lines (Ab, B16F10, and A375) of different species origin was analyzed during in vitro induced melanization in these cells. Melanin production was induced by DMEM medium characterized by a high level of L-tyrosine (a basic amino acid for melanogenesis). The biodiversity of amelanotic melanoma cells was confirmed by their different responses to melanogenesis induction; Ab hamster melanomas underwent intensive melanization, mouse B16F10 darkened slightly, while human A375 cells did not show any change in melanin content. Highly melanized Ab cells entered a cell death pathway, while slight melanization did not influence cell biology in a significant way. The rapid and high melanization of Ab cells induced apoptosis documented by phosphatidylserine externalization, caspase activation, and mitochondrial energetic state decrease. Melanoma cell type, culture medium, and time of incubation should be taken into consideration during amelanotic melanoma cell culture in vitro. L-tyrosine, as a concentration-dependent factor presented in the culture media, could stimulate some amelanotic melanoma cell lines (Ab, B16F10) to melanin production. The presence of melanin should be considered in the examination of antimelanoma compounds in vitro, because induction of melanin may interfere or be helpful in the treatment of amelanotic melanoma.

## Introduction

A malignant melanoma is a tumor that develops from melanocytes or melanoblasts (Borovanský and Riley 2011; Bertolotto 2013; Cichorek et al. 2013). It is one of the most dangerous skin cancers due to its aggressive growth, early metastatic dissemination, and resistance to treatment. A total of 2–8% of all melanomas are characterized by the absence of or a low level of melanin; this kind of melanoma is referred to as amelanotic or hypomelanotic. This rare cancer is often highly aggressive and poorly diagnosed and as a consequence has the worse prognosis (Joshi et al. 2012; Thomas et al. 2014; Ungureanu et al. 2015; Gil et al. 2019).

The main difference in cell biology between amelanotic and melanotic melanoma is based on melanogenesis, the processes of melanin synthesis controlled by tyrosinase. It has been shown that the presence of melanin influences melanoma cell biology in a significant way (Slominski et al. 2004; Wasiewicz et al. 2015; Śniegocka et al. 2018). Melanin and melanogenesis can affect functions of the epidermal cells and melanomas in a complex way (Slominski et al. 1999, 2014). Melanogenesis produces two kinds of melanin: orange-red pheomelanin and/or brown-black eumelanin. The final cell melanization is the result of a mixture of these melanins. The proportion between eumelanin and pheomelanin depends on the availability of amino acids (mainly L-cysteine) (Sugumaran 1991; Morgan et al. 2013; Panzella et al. 2014; Wasiewicz et al. 2015). Melanin formation occurs within specialized organelles known as melanosomes, which separate cytotoxic melanogenesis intermediates from cellular components (Pawelek et al. 1973; Riley 2003). The melanosomal membrane protects cells from the leakage of harmful substances (endogenous melanogenic cytotoxicity, EMC) (Miranda et al. 1984, 1994; Riley 2003; Chen et al. 2009); however, such protection could be unstable as the result of melanosomal membrane disruption that could result in melanocyte death (Lerner and Fitzpatrick 1950; Pawelek 1976; Okubo et al. 2016). The potential cytotoxic influence of melanogenesis intermediates, phenolic products and quinones (e.g., L-DOPA and DHI) was noted many years ago (Pawelek and Lerner 1978; Miranda et al. 1987; Poma et al. 1999; Hochstein and Cohen 2006). In 1978, Pawelek et al. confirmed that some melanin precursors are cytotoxic, and cells with intensive melanization could die as the result of the accumulation of these elements (Pawelek and Lerner 1978). Lerner et al. even proposed an autodestructive theory in which melanocytes are able to produce autotoxic substances (Lerner and Fitzpatrick 1950; Pawelek and Lerner 1978). Intensive melanin synthesis leads to the accumulation of toxic intermediates and increases the probability of self-destruction (Lerner and Fitzpatrick 1950; Pawelek 1976). According to other researchers, very intensive melanogenesis and overexpression of tyrosinase in melanoma is often connected with defective melanosomes, melanosomal membrane destruction, and leakage of intermediates into the cytoplasm (Miranda et al. 1994; Riley 2003). In such situations, harmful substances (e.g., quinones) are transformed into a less harmful form (e.g., by reaction with glutathione (GSH), which may be catalyzed by cytosolic glutathione-S-transferase) (Riley 2003). Melanogenesis intermediates may be considered as an internal support in antimelanoma therapy (Miranda et al. 1987).

The influence of melanogenesis and melanin presence on melanoma cell biology is still an open question. Additionally, for melanoma lines in in vitro culture, media such as RPMI1640 and DMEM are used that contain different amounts of tyrosine, the basic amino acid in melanogenesis. Thus, the aim of this study was to exam the influence of induced in vitro melanization on amelanotic melanoma cell proliferation, energy metabolism, the formation of reactive oxygen species, the cell cycle, and cell survival.

## Materials and methods

In performed experiment were used three amelanotic melanoma lines.

### Amelanotic melanoma lines

#### Bomirski hamster melanoma model

The amelanotic melanoma line (Ab) originates from the native, melanotic Ma form (appeared on hamster`s skin in a male Syrian (golden) hamster (*Mesocricetus auratus*)) by a spontaneous alteration (Bomirski 1977; Słominski et al. 1988). Professor Andrzej Bomirski indicated the block in melanosomes biogenesis (absence of premelanosomes) as the main reason of Ab cells differentiation (Bomirski 1977). The lack of melanin synthesis is accompanied by changes in many biological features, such as faster tumor growth rate, shorter animal survival, or changes in cell ultrastructure (Bomirski et al. 1988; Śniegocka et al. 2018). The amelanotic melanoma is transplanted on 3–12-month-old male Syrian (golden) hamsters by consecutive, subcutaneous injection of tumor cells every 10–12 days. Ab cells were isolated from the solid tumors by a non-enzymatic method reported previously (Cichorek et al. 2007). Cell suspension containing 90–95% of viable cells were cultured overnight before experiments to allow cells to adhere to the culture plate (time 0).

#### B16F10

B16F10 murine melanoma line was a gift from Dr. Koszalko P (Cell Biology Department of Medical University of Gdansk). It is an amelanotic melanoma line obtained from pulmonary metastasis syngeneic to C57BL/6J mice (Grygier et al. 2013).

#### A375

A375 are adherent, metastatic cells of the human amelanotic melanoma line. They have been derived from a 54-year-old female patient from metastases to the skin. Cells were provided by the American Type Culture Collection (ATCC).

### Amelanotic melanoma cells culture in media differing in L-tyrosine content

Cells of Ab, B16F10, and A375 lines were divided into two groups—the first was cultured in the RPMI-1640 (Roswell Park Memorial Institute) medium, and the second, in DMEM (Dulbecco Modified Minimal Essential Medium), both supplemented with 10% FBS and antibiotics: penicillin (100 U/ml) and streptomycin (100 μg/ml). The cultures were maintained at 37°C in 5% CO_2_. Both media are recommended for in vitro melanoma cells culture. DMEM medium contains more (72 mg/l) L-tyrosine, the basic amino acid for melanin synthesis, than RPMI (20 mg/l). Media differ also in phenylalanine level (66 mg/l in DMEM, 15 mg/l in RPMI), which could be hydroxylated into L-tyrosine in the presence of L-phenylalanine hydroxylase. DMEM, as a medium with higher L-tyrosine content, is indicated as a factor able to induce melanization in amelanotic melanoma cells (Slominski et al. 1999; Brożyna et al. 2008; Park et al. 2018). There are experimental works providing for A375 culture RPMI (Scott et al. 2007; Zhang and Wang 2019) or DMEM (Pal et al. 2014; Wang et al. 2016) media, the same referred B16F10 (Overwijk and Restifo 2000; Diawpanich et al. 2008; Yoshiura et al. 2009; Cunha et al. 2012; Burghoff et al. 2014; Potez et al. 2018). Cells were harvested after 12, 24, 36, and 48 h of incubation in media, and the number of cells, melanin content, morphological changes, cell cycle, apoptosis, ROS, energetic state were analyzed.

### Melanin content

The total amount of melanin in cells was estimated spectrophotometrically (Multiscan FC, Thermo Scientific). A total of 2×10^6^ cells were incubated with 1 N NaOH in 10% DMSO for 2 h at 60°C with shaking (Hu 2008). Samples were plated on 96-well plates, and the absorbance was measured at 450 nm. Melanin content was determined using the standard curve of synthetic melanin (Sigma Aldrich).

### Number of cells

The cell number was counted by manual method using haemocytometer (the Bürker chamber) under a microscope. The number of cells was calculated as percentage of control cells (time 0) that were assumed to be 100%.

### Cell cycle analysis

Cell cycle distribution was determined by flow cytometry method based on the DNA content in cells’ nuclei. Ethanol-fixed 1×10^6^ melanoma cells were resuspended in 1 ml of staining solution (RNAse 200 μg/ml and PI-propidium iodide, 5 μg/ml in PBS) (Darzynkiewicz et al. 1984). Then cells were incubated for 30 min at 37°C in the dark. Fluorescence was measured using a FACS Calibur flow cytometer (Becton Dickinson Immunocytometry Systems, USA). After gating out small-sized (e.g., noncellular debris) objects, 10,000–30,000 cells were collected from each sample. Results were analyzed off line using Cyflogic v.1.2.1 software.

### Morphological changes of cells

Cell morphology changes were observed by inverted light microscope (Olympus CKX31, Olympus Life Science). Additionally flow cytometry parameters as FSC (Forward Scatter) and SSC (Side Scatter) allow to follow changes of cell size and granularity respectively.

### Reactive oxygen species (ROS)

Increased ROS level can activate anti-tumorigenic signaling resulting in oxidative stress induced-cancer cell death (Darzynkiewicz et al. 1984; Wlodkowic et al. 2011). An evaluation of ROS was performed by H_2_DCFDA (2′,7′-dichlorofluorescin diacetate) staining; thus, 0.5 × 10^6^ cells were incubated with H_2_DCFDA (10 μM) for 30 min at 37°C in the dark. To create positive controls (PC), oxidative activity was stimulated with H_2_O^2^ added to probes to a final concentration about 50 μM. Fluorescence was measured using a flow cytometer, and 10,000–30,000 cells were collected from each sample. Results were analyzed offline using Cyflogic v.1.2.1 software.

### Energetic status of cells

To determine ATP and NAD concentration, frozen cells were extracted with 0.4 mol/L HClO_4_ (300 μl) and centrifuged (14,000 rpm, 10 min, 4°C). Supernatants were neutralized to pH 6 with 3 mol/L K_3_PO_4_ and centrifuged on ice after 15 min (14,000 rpm, 10 min, 4°C). The concentration of nucleotides in supernatants was measured by HPLC as described earlier (Smolenski et al. 1990). Protein precipitates were dissolved in 0.5 M NaOH and analyzed for protein concentration with the Bradford method.

### Apoptosis

Changes of the plasma membrane (phosphatidylserine and calreticulin externalization) and caspase activation as basic elements of apoptotic cell death were analyzed by a flow cytometric method. After gating out small-sized (e.g., noncellular debris) objects, 10,000–30,000 cells were collected from each sample. Results were analyzed offline using Cyflogic v.1.2.1 software.

#### Phosphatidylserine (PS) externalization assay

Early apoptotic change of plasma membrane structure, phosphatidylserine (PS) externalization, was assessed by staining cells with Annexin V and PI according to the manufacturer’s instructions (Annexin V-Fluos staining kit, Roche). The staining allows to determine populations of cells: viable (A−/PI−), early apoptotic (A+/PI−), late apoptotic (A+/PI+), and necrotic (A−/PI+).

#### Caspase activation

To estimate cells containing activated caspase FLICA test (flurochrome-labeled inhibitors of caspases) was used. In this method, fluorochrome-labeled inhibitor of caspases covalently react with reactive enzymatic center of activated caspase. We used FITC labeled pan-inhibitor of caspases VAD-FMK which detects most active caspases in a cell (Smolewski et al. 2002). A total of 0.5 × 10^6^ cells were incubated with 5 μM FITC-VAD-FMK (CaspACETM FITC-VAD-MK, Promega Probes) for 30 min. at room temperature in the dark, washed, and resuspended in PBS with 1 μg/ml of PI. Simultaneous staining with FITC-VAD-FMK and PI allows to follow dynamics of apoptotic death by distinguishing the following sequential cell stages: viable (C−/PI−), early apoptotic (C+/PI−), late apoptotic (C+/PI+) and necrotic (C−/PI+).

#### Calreticulin externalization assay

The presence of CRT in the plasma membrane characterizes substances that induce the immunogenic type of cell death (Kepp et al. 2011). CRT externalization could be a signal for immunological cells (Zitvogel et al. 2010). The presence of CRT in the plasma membrane was detected by anti-CRT antibody conjugated with PE (phycoerythrin; Abcam, Great Britain). A total of 1 × 10^6^ cells were incubated with antibody diluted 1:100 for 30 min at 4°C and analyzed in flow cytometer.

### Statistical analysis

Data are expressed as arithmetic means ± SD. STATISTICA—data analysis software system version 12 (StatSoft, 2014)—was used only for those basic statistical estimates. To avoid problems with normality and/or differences among variances for further statistical analyses only nonparametric tests were used (KyPlot v. 2.0). Among them, for trend (change of data) analysis, Jonckheere test was used; statistical significance was assumed when *p* ≤ 0.05 (*p ≤ 0,05; **p ≤ 0,01; ***p ≤ 0,001), and for differences between groups in melanin content in particular time points, U Mann-Whitney test was done, additionally.

## Results

The examined amelanotic melanoma lines reacted in different ways to incubation in DMEM medium.

### Melanin content

**Fig. 1:**
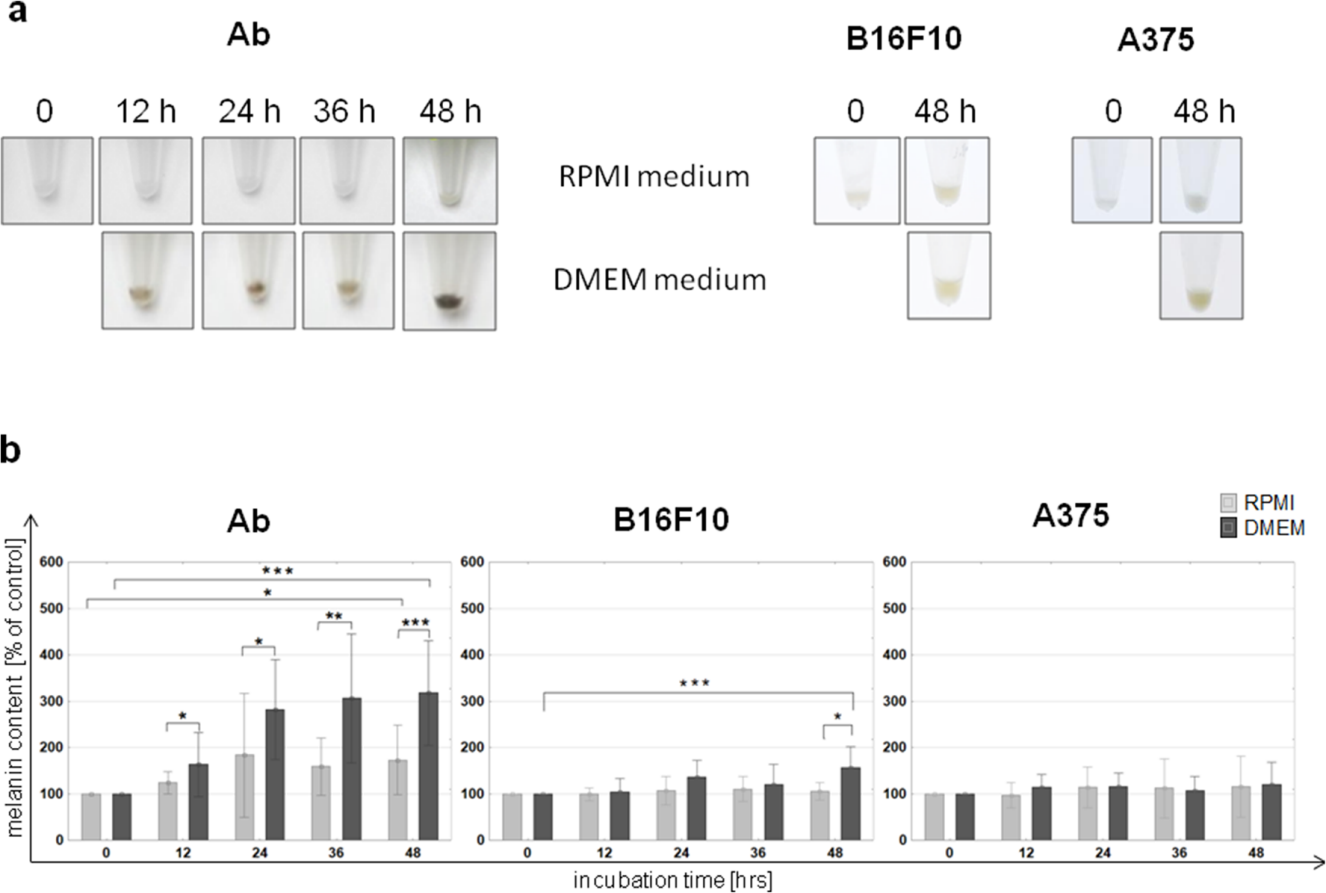
Effect of RPMI or DMEM medium on pigmentation in hamster Ab, mouse B16F10, and human A375 amelanotic melanoma cells: **a** Changes in cell pellet color and **b** changes of melanin content in cells (time 0 assumed to be 100%). Asterisk indicates a statistically significant difference between cells cultured in different media, at the same time point; *p* < 0.05, ** *p*≤0.01, *** *p*≤ 0.001. Data are related to 2 × 10^6^ cells

- Ab melanoma: Both of the used media (RPMI, DMEM) induced Bomirski Ab melanoma cell melanization although to a different degree. The RPMI medium induced slight melanization (*p* ≤ 0.05) while in DMEM, this was intensive and rapid (*p* ≤ 0.001), easily observed macroscopically as a cell pellet which darkened with time (Fig. 1a). After 48 h in RPMI, there was a 50% increase in the amount of melanin, while in DMEM, this increase was nearly 200% in comparison to time 0 (*p* < 0.05 and *p* < 0.001, respectively). The dynamic of melanin synthesis in both media was the most intensive during the first day and slowed down on the following day (Fig. 1b).
- B16F10 melanoma: Melanin content increased only in B16F10 cells cultured in DMEM (*p*≤0.01). After the first day of incubation, the amount of melanin increased by over 30% and reached more than 50% during the following day in comparison with time 0; thus, the intensity of melanization was the highest during the first day (Fig. 1b).
- A375 melanoma: A375 cells did not undergo melanization, regardless of the time of incubation or media used. During the experiment, the content of melanin did not change in comparison with the initial value (Fig. 1b).

### Cell proliferation

**Fig. 2:**
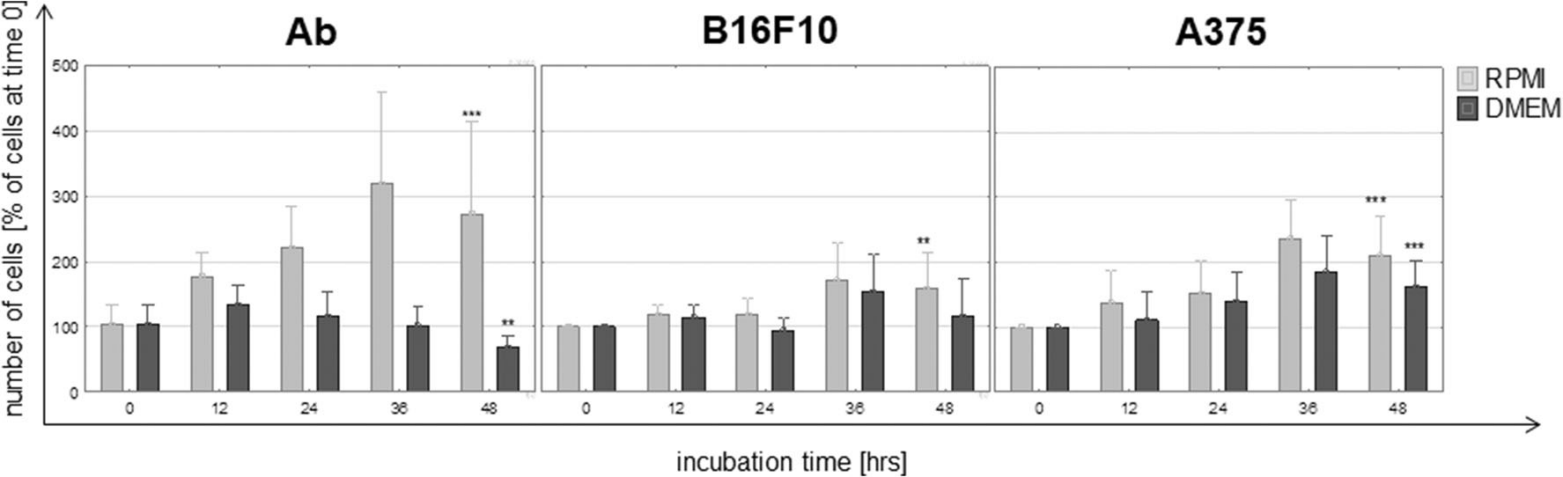
Proliferation of amelanotic melanoma cells in hamster Ab, mouse B16F10, and human A375 during culture in RPMI or DMEM media (time 0 assumed to be 100%). Asterisk indicates a statistically significant change compared with time 0; * *p*≤0.05, ** *p*≤0.01, *** *p*≤ 0.001, nonparametric Jonckheere trend test

- Ab melanoma: Ab cells in RPMI medium proliferated constantly (*p* <0.001). The highest increase (over 200%) was noticed after 36 h, but after an additional 12 h, the number of cells decreased slightly (Fig. 2). In DMEM medium, an increase in the number of cells was observed only during the first 12 h (by over 30%) but was lower than in RPMI. After that time, the number of cells gradually decreased (*p*≤0.01) (Fig. 2).
- B16F10 melanoma: The number of B16F10 cells after 2 days in RPMI increased by 50% (*p*≤0.05). In DMEM, the cell number did not change significantly.
- A375 melanoma: A375 cells cultured in both tested media doubled their number (*p* < 0.001) after 2 days.

DMEM induced melanization in two of the three examined amelanotic melanomas, hamster Ab and mouse B16F10. Thus, we decided to analyze how this in vitro induced melanization affected the cell biology (cell cycle, metabolism, ROS production) of these amelanotic melanoma lines.

### Cell cycle analysis

**Fig. 3:**
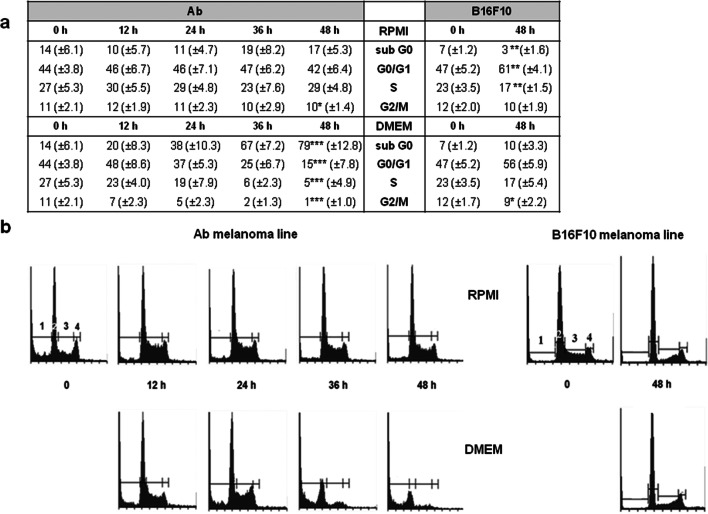
Ab and B16F10 melanoma cell cycle analysis. **a** Percentage of cells in each cycle phase presented as means ± SD of 8 experiments for Ab and 5 for B16F10. Asterisk indicates a statistically significant change compared with time 0; * *p*≤0.05, ** *p*≤0.01, *** *p* ≤ 0.001, nonparametric Jonckheere trend test. **b** Representative histograms of DNA content in Ab and B16F10 melanoma cells cultured in RPMI or DMEM media. Markers: 1: subG0 (cells with decreased DNA content e.g., apoptotic bodies), 2: G0/G1 phase, 3: S phase, and 4: G2/M phase

- Ab melanoma: At time 0, Ab cells comprised 44 % in the G0/G1, 27% in the S, and 11% in the G2/M phases (Fig. 3a). After 48 h of incubation in DMEM, the percentage of cells in each phase dramatically decreased to 15% in the G0/G1 phase, 5% in the S, and 1% in the G2/M (*p* ≤ 0.001) phase (Fig. 3a). These changes were also accompanied by a significant increase in the number of cells in the subG0 area (damaged cells with decreased DNA content). Finally, after 48 h nearly 80% of the Ab cells accumulated in this area (*p* ≤ 0.001) (Fig. 3a, b, marker 1). The above observation coincides with the increasing amount of melanin that reached over 300% in DMEM. The decreasing viability accompanying the progress of pigmentation suggests that the induced melanogenesis in Ab amelanotic melanoma cells leads to cell death. In RPMI, there were no significant statistical changes in the number of cells in each cell cycle phase.
- B16F10 melanoma: It was noted that B16F10 cells in RPMI accumulated in the G0/G1 phase as their number increased from 47 to 61% (*p*≤0.01; Fig. 3a, b, marker 2). The percentage of these cells in the S+G2/M and subG0 phases slightly decreased from 35 to 27% and from 7 to 3 % (*p*≤0.01), respectively (Fig. 3a). Cells cultured in DMEM showed a decrease in the G2/M phase from 12 to 9% (*p*≤0.05, Fig. 3a). The number of cells in the G0/G1 and S phase did not change significantly (G0/G1 from 47 to 56 %; S from 23 to 17 %; Fig. 3a).

### Morphological changes

Observations of cells with a light microscope and using flow cytometry (parameters: FSC as cell diameter, SSC as granularity) documented morphological changes during melanogenesis.

**Fig. 4:**
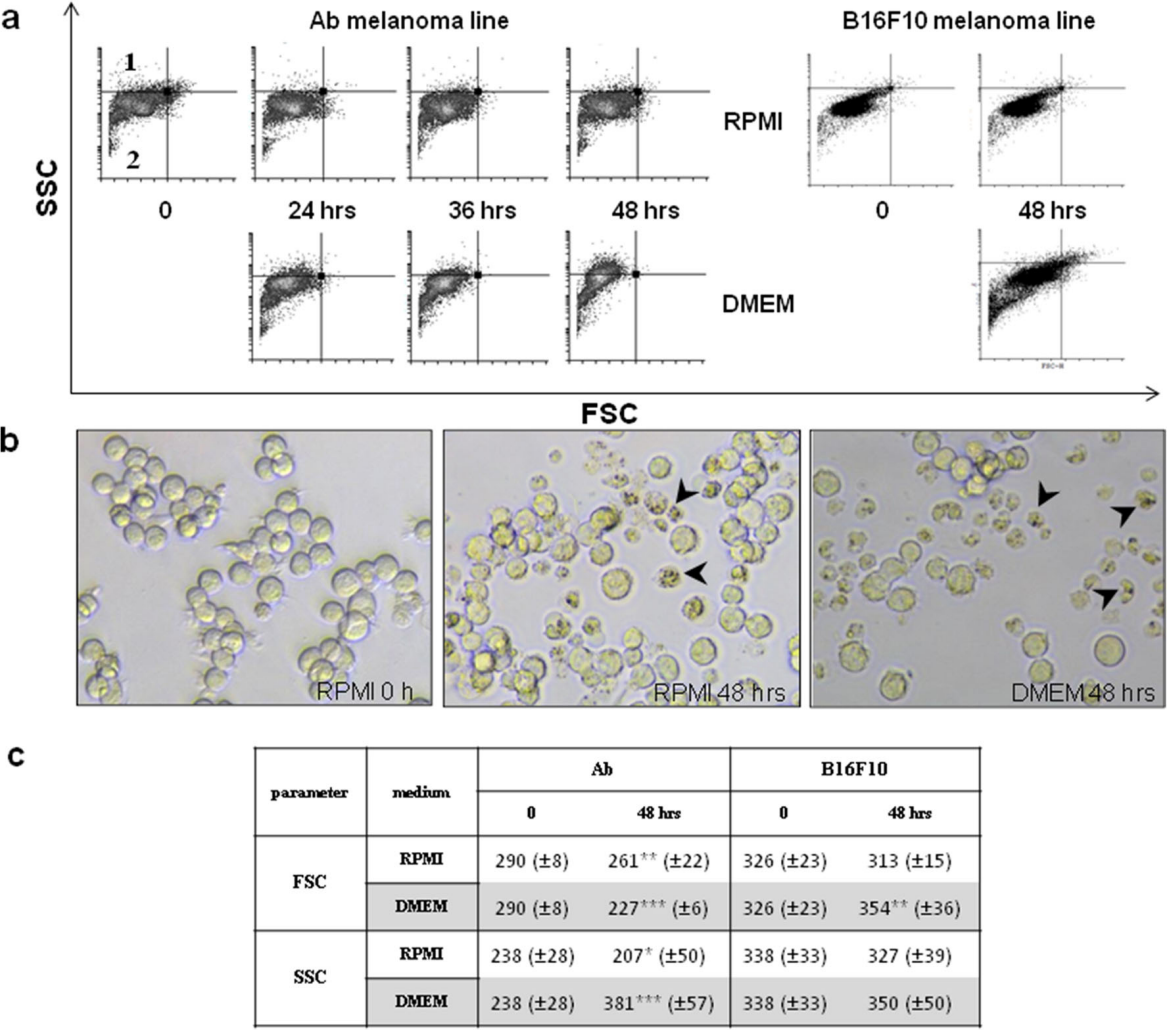
Morphological changes of amelanotic cells during ongoing melanization. **a** Size and granularity changes in Ab and B16F10 cells cultured in RPMI or DMEM media. Cells localized in quadrant 1 are characterized by high SSC (granularity) and low FSC (diameter), while in quadrant 2 are cells with low FSC and SSC. **b** Morphological changes in an Ab cell observed by light microscope at time 0 and after 48 h in RPMI or DMEM media. Arrowheads indicate darkening, shrunk cells with visible melanin granules inside. **c** Changes in FSC and SSC parameters (including statistical significance: * *p*≤0.05, ** *p*≤0.01, *** *p* ≤ 0.001) in amelanotic melanoma cell lines: Ab and B16F10 cultured in RPMI or DMEM media (5 experiments for each cell line)

- Ab melanoma: It was observed via flow cytometry that FSC and SSC parameters of Ab cells cultured in RPMI decreased (*p*>0.01), while in DMEM, FSC also decreased (*p*>0.001) but SSC increased (*p*>0.001) (Fig. 4c). Changes in DMEM were the consequences of the appearance of melanin granules in cells (SSC increase) and cell shrinkage (FSC decrease) (Fig.4a; 48 h, quadrant 1]. Morphological changes were also documented by the light microscope (Fig. 4b). Cells undergoing melanization (melanin pigment was visible inside the cells as dark dots) became smaller and detached from the bottom of the culture plate. In RPMI, most cells remained morphologically as they were at the beginning; round, lustrous, and the same size. Only a small number of the cells underwent melanization, while in DMEM, these melanizing cells dominated.
- B16F10 melanoma: In DMEM culture, FSC and SSC parameters increased with incubation time (Fig. 4a, c). Additionally, cells formed more dendrites, making them strongly adhere to the bottom of the dish culture.

### Reactive oxygen species

**Fig. 5:**
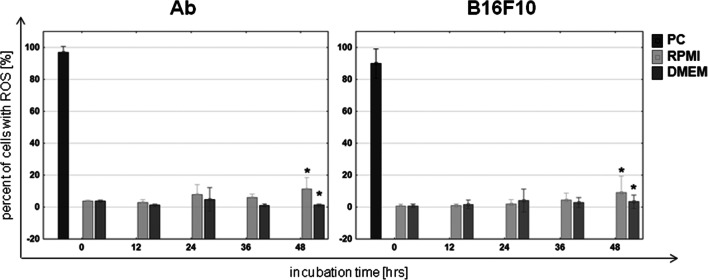
Content of cells with reactive oxygen species (ROS) in Ab and B16F10 melanoma cultured in RPMI or DMEM. Cells treated with H_2_O_2_ to a final concentration of 50 μM serve as a positive control (PC) of ROS presence. Asterisk indicates a statistically significant change compared with time 0; *p*≤0.05, nonparametric Jonckheere trend test; means data ± SD of 4 independent experiments for each cell line

- Ab melanoma: Ab cells cultured in RPMI medium showed an increasing rate of cells with ROS (*p*≤0.05). This increased from 3.8% at time 0 to 13.9% after 48 h (Fig. 5). In DMEM medium, the Ab line was characterized by a decreasing percentage of cells with ROS (*p*≤0.05), from the initial 3.8 to 1.3% at the end of the experiment. Such low ROS values indicated that these rather do not participate in the observed Ab cell death during induced melanization.
- B16F10 melanoma: The rate of B16F10 cells with ROS increased with the extension of the time of incubation in RPMI (*p*≤0.05). In DMEM medium, the percentage of ROS increased, but only during the first day (*p*≤0.05) (Fig. 5).

### Cell energetic state: ATP and NAD levels

#### Ab melanoma

**Fig. 6:**
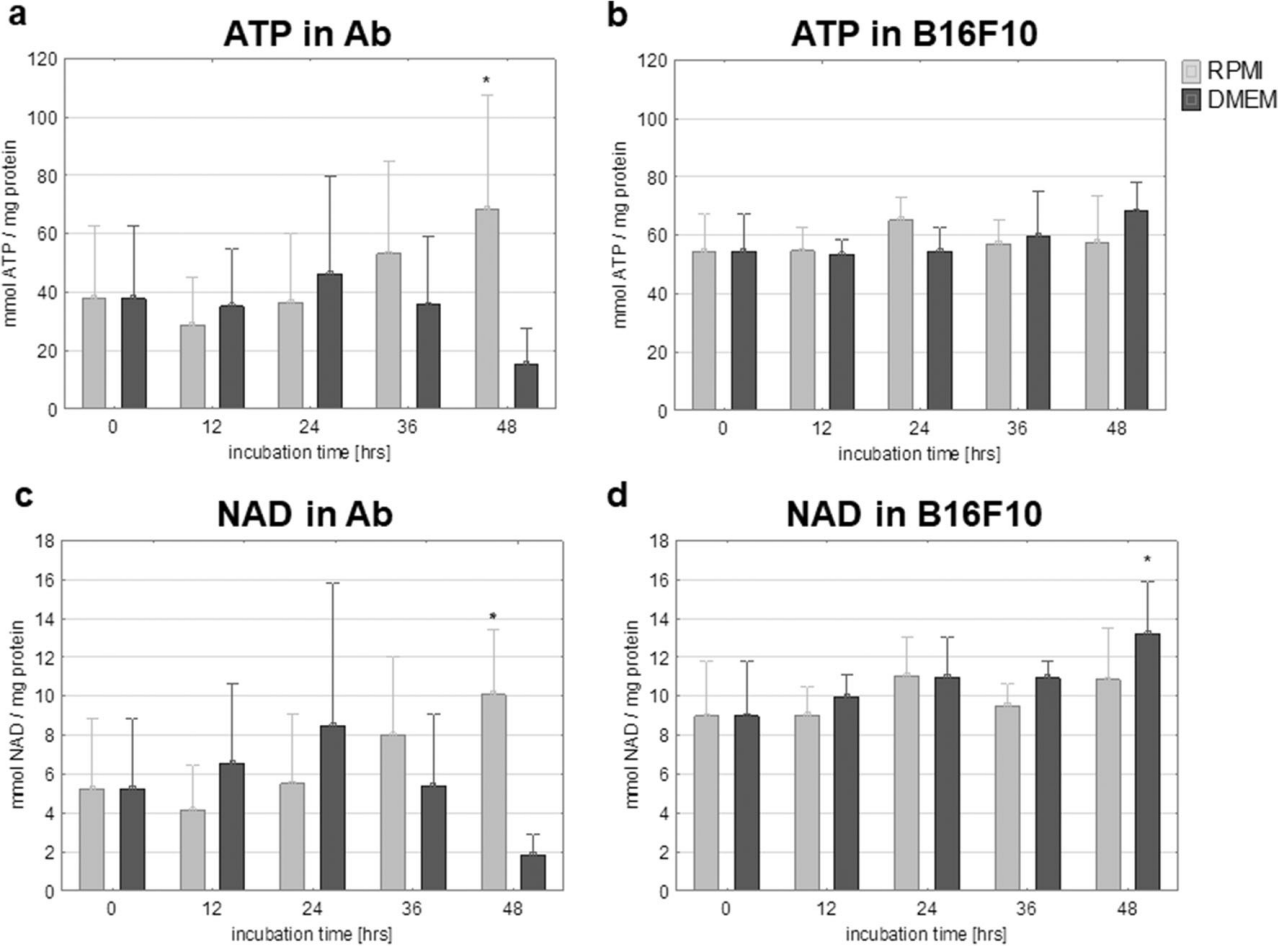
Changes of ATP content in **a** Ab cells and **b** B16F10 cells and NAD in **c** Ab cells and **d** B16F10 cells. Asterisk indicates a statistically significant change compared to time 0; *p*≤0.05, nonparametric Jonckheere trend test; means data ± SD of 4 independent experiments for B16F10 and 3 for Ab

- ATP: In cells of the Ab line, the amount of ATP significantly increased from ~38 to 69 mmol/mg of protein (*p*≤0.05) with prolonged incubation time in RPMI. In DMEM medium, the content of ATP decreased from ~38 to 15 mmol/mg of protein on the second day of incubation (Fig. 6a). The decrease in ATP occurred at the same time as the intensive (threefold) increase in melanin content and accumulation of cells in the subG0 phase (cell damage).
- NAD: Changes in NAD were analogous to changes in ATP. The level of NAD in Ab cells increased with the incubation time in RPMI from 5 to 10 mmol/mg of protein (*p*≤0.05) (Fig. 6c). In DMEM medium, NAD content increased up to 8 mmol/mg of protein only on the first day, and on the second day this value decreased to ~2 mmol/mg of protein (Fig. 6c).

#### B16F10 melanoma

- ATP: The concentration of ATP in B16F10 cells in both RPMI and DMEM medium did not change significantly, although after 48 h, a slight increase from 55 to 68 mmol/mg of protein was observed (Fig. 6b). During this time, an increase in melanin was observed.
- NAD: NAD level in B16F10 cells cultured in DMEM increased from 9 to 13 mmol/mg of protein (*p*≤0.05). In RPMI, there was also an increase (from 9 to ~11 mmol/mg of protein), but it was statistically insignificant (Fig. 6d).

### Apoptosis analysis

Cell cycle results indicated that Ab cells cultured in DMEM entered a death pathway. To determine the type of cell death (Wlodkowic et al. 2011; Galluzzi et al. 2018), we decided to estimate the externalization of phosphatidylserine and calreticulin as markers of the plasma membrane changes and caspase activation, the main elements of apoptotic cell death.

#### Ab melanoma

**Fig. 7:**
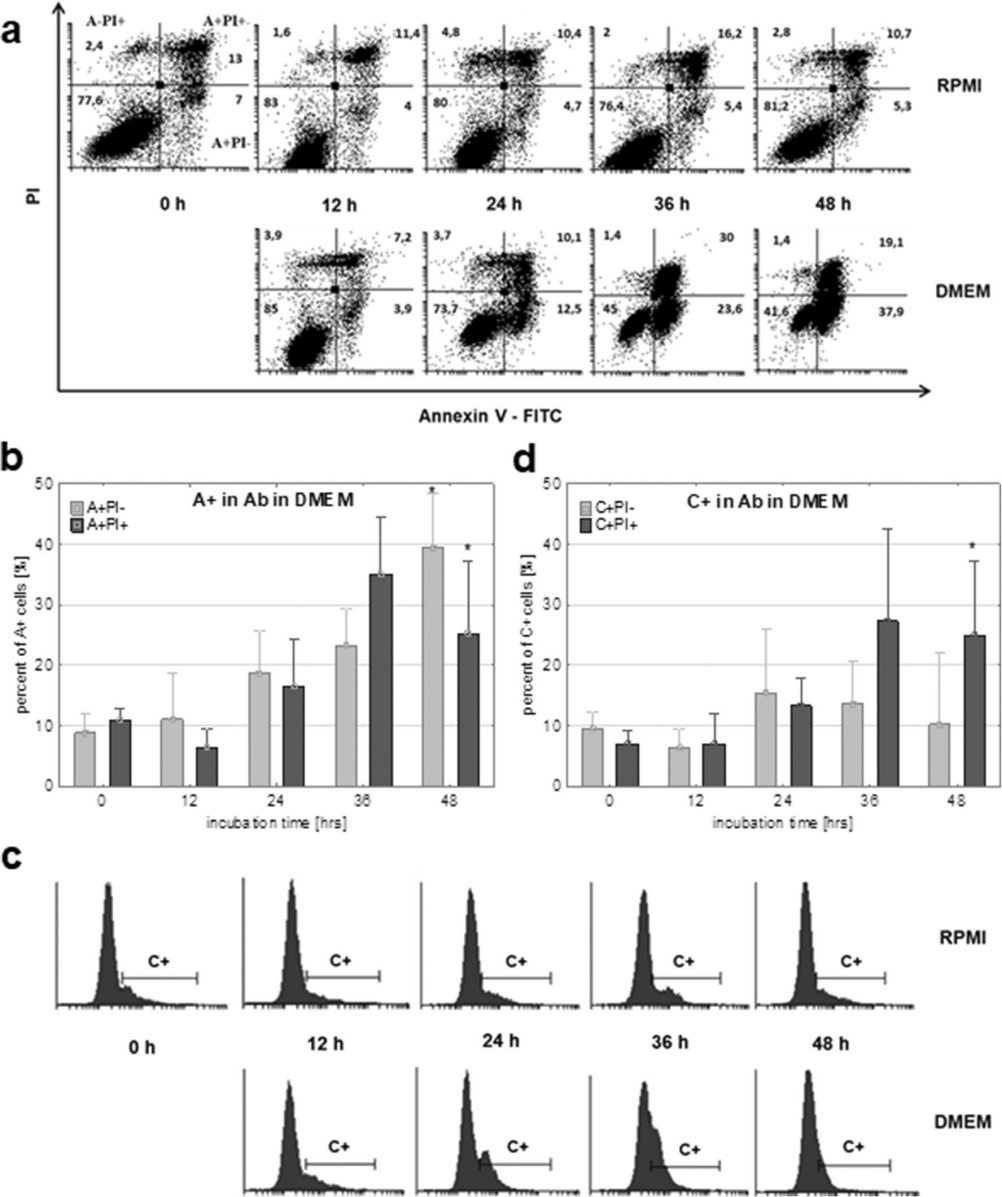
Apoptotic cell death analysis in Ab cells cultured in RPMI or DMEM. **a**, **b** Externalization of phosphatidylserine in the plasma membrane: **a** representative flow cytometry dot plots results with annexin positive cells (A+PI−, A+PI+); **b** percentage of annexin positive cells (A+PI−, A+PI+) presented as means ± SD of 6 independent experiments. **c**, **d** Caspase activation in Ab cells: **c** representative flow cytometry histograms with caspase positive cells (C+); **d** percentage of Ab cells with activated caspases presented as means ± SD of 7 independent experiments. A+PI− and C+PI−, early apoptotic cells; A+PI+ and C+PI+, late apoptotic cells; A−PI+, necrotic cells; A+, all annexin-positive cells; C+, cells with activated caspases. Asterisk indicates a statistically significant change compared with time 0; *p*≤0.05, nonparametric Jonckheere trend test

- Phosphatidylserine (PS) externalization assay: Externalization of phosphatidylserine (PS) is a hallmark of the changes in the cell surface during apoptosis. Binding of Annexin V to Ab cells showed a higher content of cells with PS externalization in DMEM. The Annexin-positive cells (A+; a sum of A+PI− and A+PI+) increased from 19 (±4.3)% to 64 (±14.8)% over 48 h (*p* ≤ 0.001) (Fig. 7a, b). Among them, the number of early apoptotic cells increased constantly, reaching 40% at the end (A+PI−, *p*≤0.001), while the number of late apoptotic cells increased over 36 h and then decreased ([A+PI+, *p*≤0.001] Fig. 7b). After 36 h, late apoptotic cells (A+PI+) dominated, and after an additional 12 h, early apoptotic cells were dominant (A+PI−). The level of Annexin-positive cells in RPMI did not change significantly (results not shown).
- Caspase activation: Analysis of activated caspases in Ab cells cultured in DMEM demonstrated externalization changes similar to PS. The caspase-positive cells (C+; a sum of C+PI− and C+PI+) increased, achieving their maximum level of 45% after 36 h (Fig. 7c, d). Population analysis indicated an almost constant level of early apoptotic cells (10%; C+PI−) and an increase of late apoptotic cells (C+PI+, *p*≤0.001). The content of necrotic cells (C−PI+) also increased from 3 to over 30% at the end of the experiment, while in RPMI, it remained unchanged (from 3 to 5%; data not shown).
- Calreticulin (CRT) externalization assay: Incubation of Ab cells in both media did not induce calreticulin translocation to the plasma membrane of Ab cells and other examined amelanotic melanoma lines.

Analysis of processes related to apoptosis (caspases activation, phosphatidylserine externalization) showed that only in Ab amelanotic melanoma cells did the induction of melanin production initiate the apoptosis of these cells.

## Discussion

Melanoma as a dangerous and fast-spreading skin cancer remains a substantial epidemiological problem (Gil et al. 2019). Amelanotic melanoma is a rare form of melanoma characterized by a low or absent melanin content. Although the melanization process of amelanotic melanoma cells has been observed in vitro for many years, its influence on melanoma biology is not well understood (Cheung et al. 2012).

One of the best-known melanoma cell lines are mouse B16 cells, characterized by their different capacity for melanin synthesis and proliferative potential. The first observations in the 1970s showed that induced-melanogenesis in B16 amelanotic cells affected their size (the cells became larger) and decreased proliferation during differentiation into melanotic cells (Kreider and Schmoyer 1975). It was also observed that amelanotic Ab melanoma culture in MEM medium (minimal essential medium, 36 μg/ml L-tyrosine) underwent melanization, and the cells detached from the bottom of the dish culture and lost ability to proliferation (Słomiński and Bomirski 1985). Hamster Ab melanoma cells, despite the lack of melanogenesis, are able to produce melanosomes and to synthesize proteins involved in melanin production (tyrosinase-related protein 1-TRP1, tyrosinase-related protein 2 - TRP2), including tyrosinase (Slominski and Costantino 1991). These cells after transfer to in vitro culture regain the capacity for melanin synthesis (Słominski 1983; Słomiński and Bomirski 1985). Słomiński show that the detached heavily pigmented cells after transplantation back to hamsters induce amelanotic melanomas indicating that melanogenic path and the apoptosis paths are reversible in vivo (Słomiński 1985) .

One of the methods used to induce melanogenesis is the application of amelanotic melanoma cells into an appropriate cell culture medium. Media differ in the content of amino acids, glucose, and metal ions; however, in terms of melanogenesis, the L-tyrosine level is the most important (Slominski and Ermak 1999; Brożyna et al. 2008). L-tyrosine has an effect at various stages of melanogenesis, e.g., the translocation of tyrosinase from the Golgi apparatus to melanosomes, the activation of proteins involved in melanogenesis, the formation of premelanosomes, and maturation of melanosomes (Slominski et al. 2012). The stimulatory properties of tyrosine refer only to the L-tyrosine stereoisomer; D-tyrosine inhibits melanization by tyrosinase activity suppression (Słominski et al. 1988; Park et al. 2018).

In the performed experiment, amelanotic melanomas from different species, hamster (Ab), mouse (B16F10) and human (A375), were incubated in media with different L-tyrosine levels. The medium with the higher level of tyrosine (DMEM) induced melanization of examined amelanotic melanoma cells but to a different degree.

The intensive melanization of Ab melanoma in DMEM and slight melanin production in RPMI seemed to stress L-tyrosine-dependent production of melanin in these amelanotic melanoma cells. These results confirmed the earlier observation of Miranda et al. that the amelanotic phenotype undergoes faster melanization at higher levels of L-tyrosine (Miranda et al. 1994).

In B16F10, melanogenesis was induced only in cells cultured in DMEM, but the process was less intensive than in Ab cells. DMEM medium was totally ineffective in human amelanotic melanoma cells A375. The heterogenous reaction of amelanotic melanoma cells to L-tyrosine-induced melanization stressed that the lack of melanin may be the result of a different mechanism. Additionally, our previous results documented that Ab and B16F10 cells have an active tyrosinase, the basic enzyme of melanogenesis, while A375 do not (Skoniecka et al. 2018). According to Kudugunti et al., the selective toxicity of some substances on amelanotic cells depends on the tyrosinase functionality (Kudugunti et al. 2010).

Induced melanogenesis may change the biology of amelanotic melanoma cells. Ab cells in DMEM were the amelanotic melanoma cells which exhibited the most darkening. The presence of melanin granules and tyrosinase activation were observed in Ab cells cultured in DMEM in our earlier studies (Skoniecka et al. 2018). Morphological changes, decreasing cell numbers and significant displacement of cells from all cell cycle phases to the subG0/G1 fraction indicated Ab cell death. Although melanin presence increased in DMEM, the level of ROS-positive cells was very low. Thus, it seemed that it was not the basic reason for the observed increased subG0/G1 fraction. Melanogenesis influenced the energetic metabolism of Ab cells. Thus, the ATP level in Ab cultured in RPMI increased, which can be explained by ongoing proliferation, requiring mitochondrial activity. But ATP in the same cells incubated in DMEM increased during the first day and decreased the following one, as cells were dying. The substantial increase in melanin content, activation of caspases, and externalization of phosphatidylserine in cells all require energy (Scislowski and Slominski 1983; Zamaraeva et al. 2005).

B16F10 cells cultured in DMEM medium underwent melanization at a similar rate to Ab cells in RPMI (Fig. 1a, b). Slight melanization neither had a harmful influence nor did it change their biology in a significant way (Figs. 2b and 3a, b). The weaker increase of ROS level in B16F10 cells cultured in DMEM could be explained by the scavenging properties of melanin (Menon et al. 2009). Cunha et al. interpret the slower ROS increase after longer melanogenesis stimulation as cell cycle arrest in G1 phase (Cunha et al. 2012). A slight increase of NAD during B16F10 cells melanization has been also noticed by others (Liu Y. 2009). The presence of tyrosinase protein was confirmed in both Ab and B16F10 melanoma cells (Skoniecka et al. 2018). Our observation did not confirm Kreider and Schmoyer’s results which showed that ongoing melanization in B16 cells decreased proliferation in a significant way (Kreider and Schmoyer 1975). Our results showed that induced melanization caused a slight increase in the G0/G1 phase. A similar observation was made by Meira et al. who concluded that synthesis of the pigment in B16-F10 cells results in a cell cycle arrest and induction of a quiescent state, which could be a mechanism of resistance against cellular damage (Meira et al. 2017). According to Pinon et al., cells are able to trigger a resistance mechanism to delay death promoted by melanin synthesis (Pinon et al. 2011)*.*

A375 cells remained amelanotic throughout the experiment despite the high level of L-tyrosine in DMEM. None of the examined media stimulated the melanin production (Fig. 1c) nor influenced the cell biology of the A375 cell line.

Summarizing the results of amelanotic melanoma cell melanization induced by media dedicated to melanoma culture, we can conclude that melanization influences amelanotic melanoma cell biology in different ways. In the Ab melanoma line, intensive melanization leads to cell death. On the other hand, slight melanization in Ab culture in RPMI and B16F10 in DMEM does not influence their biology in a significant way.

### Melanization of amelanotic melanoma induces cells death by apoptosis

Initial and preliminary macroscopic (Fig. 4b) and cytometric observation (↓FSC, ↑SSC) (Fig. 4a, c) with decreasing cell numbers (Fig. 2) suggested apoptotic changes (cells shrinkage, condensation of nucleus, cytoplasmic membrane dehydration (Wlodkowic et al. 2011)) and the presence melanin granules within cells (Fig. 4a, b).

Cell cycle analysis highlighted the relationship between examined cell death and the melanization process (Fig. 3). Intensive pigment production was detrimental for Ab cells. Similar results but with Cloudman S-91 melanoma cells were explained by other researchers by the strong stimulation of melanin synthesis which leads to the accumulation of toxic compounds (tyrosine, DOPA, etc.) and an increase in the likelihood of cellular self-destruction (Lerner and Fitzpatrick 1950; Pawelek 1976). It was shown that during melanization, cytotoxic and genotoxic compounds such as quinones, semi-quinones, superoxide anion (O_2_^−^), and hydrogen peroxide (H_2_O_2_) can be released to the medium (Miranda et al. 1994). Melanosomes in melanoma are altered and allow cyto-genotoxic compounds (L-tyrosine, L-DOPA) to be released (Miranda et al. 1994). Indoles (e.g., the DHIC) have been shown to exhibit genotoxic activity by direct influence on the DNA bases (Pawelek and Lerner 1978; Miranda et al. 1987).

Cytometric analysis of apoptotic parameters: Externalization of phosphatidylserine (up to 64% of cells) and caspase activation (up to 45% of cells) ultimately confirmed apoptosis as the main pathway of cell death. Activation of caspases is the initiating stimuli to the final demise of the cell, inhibiting autophagy at the same time (Polewska 2012). Both of these parameters increasing over a short time indicated the rapidity and significant intensity of apoptosis, and its relation to the cytotoxic influence of the melanization process in progress on at the same time. The kinetics of cell death is individual; apoptosis from initiation to completion can transpire in only a few hours (Elmore 2007), and in the case of Ab, it took 2 days (Fig. 3). Melanogenesis in Ab cells did not cause calreticulin presence in the plasma membrane, which indicated the lack of so-called immunological cell death. This type of cell death was documented for different cells and different compounds (Zitvogel et al. 2008; Garg et al. 2017).

The above results suggest that the intensity of melanogenesis could be the important factor of melanoma cell viability. The intensity of melanogenesis in the hyperpigmented melanoma cells is often associated with the formation of defective melanosomes, which causes leakage of intermediates through discontinuous melanosomal membranes (Miranda et al. 1994; Riley 2003). The intensive melanin production and fast melanosome formation in amelanotic Ab cells seem to influence the biology of these cells. Chen et al. suggest that only stage IV melanosomes are associated with the development of EMC (endogenous melanogenic cytotoxicity) in melanotic melanoma cells (Chen et al. 2009). The way to overcome the EMC is to export the melanosomes, which took place in Ab cultured in RPMI (Chen et al. 2009). Extrusion of harmful substances outside the cell in DMEM was ineffective because of the high intensity of melanogenesis. The formation of melanosomes is probably involved in EMC detoxification (Chen et al. 2009). It is worth stressing that apoptosis was observed only in amelanotic cells undergoing intensive melanization. In RPMI medium, slight pigmentation has no significant impact on cell biology. The harmful effect of excessively fast and intensive melanogenesis seems to be confirmed by an earlier publication dealing with the melanization of Ab cells in MEM medium, which was slower and did not mention a toxic influence (Słomiński and Bomirski 1985).

Measurement of reactive oxygen species in cells showed that ROS has no impact on Ab cell death. The level of ROS-positive cells was very low at 4% and decreased with ongoing melanization, which may be connected with the appearance of melanin, which has antioxidant properties and is a free radical scavenger (Menter and Willis 1997; Dadachova and Casadevall 2008; Brożyna et al. 2008; Wang et al. 2011).

## Conclusions

The type of culture medium, time of incubation, and melanoma cell type should be taken into consideration during amelanotic melanoma cell culture in vitro. L-tyrosine, as a concentration-dependent factor, may stimulate some amelanotic melanoma cell lines (Ab, B16F10) to melanin production, but not all of them (A375). Induced melanization can influence cells differently, e.g., it may launch cells onto an apoptotic pathway via cytotoxic intermediates (Ab cells) or have no impact on their biology (B16F10).
